# Spatial-Sequential Working Memory in Younger and Older Adults: Age Predicts Backward Recall Performance within Both Age Groups

**DOI:** 10.3389/fpsyg.2016.01514

**Published:** 2016-10-04

**Authors:** Louise A. Brown

**Affiliations:** School of Psychological Sciences and Health, University of StrathclydeGlasgow, UK

**Keywords:** cognitive aging, ageing, spatial-sequential working memory, spatio-sequential, visual-spatial, visuo-spatial sketchpad, central executive attention, Corsi blocks test

## Abstract

Working memory is vulnerable to age-related decline, but there is debate regarding the age-sensitivity of different forms of spatial-sequential working memory task, depending on their passive or active nature. The functional architecture of spatial working memory was therefore explored in younger (18–40 years) and older (64–85 years) adults, using passive and active recall tasks. Spatial working memory was assessed using a modified version of the Spatial Span subtest of the Wechsler Memory Scale – Third Edition (WMS-III; Wechsler, 1998). Across both age groups, the effects of interference (control, visual, or spatial), and recall type (forward and backward), were investigated. There was a clear effect of age group, with younger adults demonstrating a larger spatial working memory capacity than the older adults overall. There was also a specific effect of interference, with the spatial interference task (spatial tapping) reliably reducing performance relative to both the control and visual interference (dynamic visual noise) conditions in both age groups and both recall types. This suggests that younger and older adults have similar dependence upon active spatial rehearsal, and that both forward and backward recall require this processing capacity. Linear regression analyses were then carried out within each age group, to assess the predictors of performance in each recall format (forward and backward). Specifically the backward recall task was significantly predicted by age, within both the younger and older adult groups. This finding supports previous literature showing lifespan linear declines in spatial-sequential working memory, and in working memory tasks from other domains, but contrasts with previous evidence that backward spatial span is no more sensitive to aging than forward span. The study suggests that backward spatial span is indeed more processing-intensive than forward span, even when both tasks include a retention period, and that age predicts backward spatial span performance across the adult lifespan, within both younger and older adulthood.

## Introduction

An important factor in spatial working memory performance is the degree of active processing involved in the task (Cornoldi and Vecchi, 2003). Passive storage involves retaining information which has not been modified after encoding, while active processing requires transforming, manipulating, or integrating information. Cornoldi and Vecchi (2003) argued that the degree of processing must be conceived along an activity continuum. Visuo-spatial tasks which require storage and retrieval do require some active processing in the form of rehearsal. However, the degree of active processing is very low compared with a task which additionally requires active manipulation of the information before the participant can provide the appropriate response. Multiple component models of working memory (e.g., Baddeley and Hitch, 1974; Logie, 1995, 2003, 2011; Baddeley, 2000, 2007, 2012) conceive of such a distinction between storage and processing. These models comprise specialized verbal and visuo-spatial storage components (the *phonological loop* and *visuo-spatial sketchpad*, respectively), as well as domain-general processing capacity (the *central executive*). Specifically regarding storage and processing in the visuo-spatial domain, Logie’s model of working memory comprises a passive visual store component, which temporarily retains visual detail (*visual cache*), and an active spatial rehearsal mechanism (*inner scribe*), which stores spatial-sequential codes. The inner scribe can also refresh the contents of the visual store, which is otherwise subject to rapid (2 s) decay (Logie, 2011). This active processing comes at a cognitive cost, however, and draws upon the capacity of the central executive (see also Rudkin et al., 2007; Vandierendonck, 2016).

Fluid cognitive abilities such as working memory are particularly vulnerable to age-related decline, and have been shown to be subject to linear decline throughout the adult lifespan, often from the early 20s (Park et al., 2002; Logie and Maylor, 2009; Johnson et al., 2010). Regarding the potential role of active, central executive processing in age-related decline, previous research has suggested that older adults exhibit specific deficits when active information processing is involved, while age differences are relatively minimal in passive tasks (Phillips and Hamilton, 2001; Bopp and Verhaeghen, 2005; Braver and West, 2008). Vecchi et al. (2005; see also Vecchi and Cornoldi, 1999; Mammarella et al., 2013) showed that active working memory tasks, in both the visuo-spatial and verbal domains, are subject to greater age-related deficits, and that age effects are typically seen earlier in the lifespan for active tasks relative to passive ones.

One validated task for assessing the performance of spatial-sequential working memory (Logie, 2011) is the Corsi blocks task (Milner, 1971; Corsi, 1972; De Renzi and Nichelli, 1975; Smyth and Scholey, 1994; Berch et al., 1998; Della Sala et al., 1999). Often described simply as *spatial span*, the task involves presenting a series of spatial sequences and asking participants to recall them either immediately or following a maintenance period. The sequences take the form of movements between various spatial locations. They may be presented to participants either via a computer screen, or using a board featuring an array of blocks that are tapped by the researcher. The latter is the case for the Spatial Span subtest of the Wechsler Memory Scale (3rd Edition; WMS-III, Wechsler, 1998). Particularly with a maintenance period inserted after presentation and before recall, the task requires active rehearsal of the information (Cornoldi and Vecchi, 2003; Logie, 2011).

The degree of active processing involved in the spatial span task is amenable to manipulation, however, with a typical comparison being whether sequences are recalled either in the same order as the researcher (forward) or the opposite order (backward). Given the above findings comparing passive vs. active working memory tasks, one could predict greater age-related decline in a backward recall version of the spatial span task, compared with forward recall. However, the findings in this respect have been mixed. For example, Hester et al. (2004) investigated the effect of age on forward and backward spatial span and the age-related decline in performance was equivalent in both measures. Similarly, Wilde et al. (2004) analyzed data from the WMS-III (Wechsler, 1997) standardization sample (*n* = 1,250). While forward recall was performed better than backward recall overall in this study, the difference between the two recall types was not enhanced in older age. The authors concluded that backward spatial recall is no more age-sensitive than forward recall (see also Myerson et al., 2003). Indeed, Hester et al. proposed that the central executive component of working memory is recruited for successful performance in both versions of the task. This could be supported by the argument that spatial-sequential working memory, even in forward recall, requires central executive processing (Vecchi and Richardson, 2001; Logie, 2011).

In younger adults, Vandierendonck et al. (2004) compared the difficulty of forward and backward spatial span and found no consistent effect of recall format (see also Wilde and Strauss, 2002, for a clinical sample). However, a targeted interference paradigm was used across varying sequence lengths, designed to disrupt specific forms of processing during encoding and to determine the underlying processes involved. Concurrent spatial tapping was intended to disrupt spatial processing, while random interval generation was performed in order to suppress central executive functioning. Vandierendonck et al. (2004) showed that active spatial processing was required throughout the task, even at lower levels of complexity, demonstrating the critical nature of this processing resource (Vecchi and Richardson, 2001; Hamilton et al., 2003). However, additionally, specifically when reaching span and supra-span levels of complexity, domain-general central executive resources were increasingly employed at the most challenging levels of the task (Vandierendonck et al., 2004; see also Hamilton et al., 2003; Thompson et al., 2006; Logie, 2011). Importantly, though, both forward and backward recall drew upon central executive resources (Rudkin et al., 2007; Vandierendonck, 2016).

Clearly, there has been debate in the literature regarding the extent of active processing required by different spatial-sequential working memory tasks, and particularly regarding the possibility that the more active backward spatial span task is especially impaired by aging. The current study assessed whether or not a differential age-related decline would be evident between conditions of relatively passive and active recall (forward vs. backward spatial span). Phase one assessed recall of sequences in the same order as presentation and required no manipulation of the material, only active spatial rehearsal. Phase two of the task, on the other hand, assessed recall of sequences in the reverse order of presentation and therefore required active rehearsal as well as active manipulation of the information prior to recall. If aging differentially affects more active spatial working memory tasks, then a greater age-related deficit would be predicted when backward recall is performed.

Regarding the potential for differential use of visuo-spatial sketchpad resources by younger and older adults, a targeted interference paradigm was additionally employed in the current study. This was to assess the extent to which younger and older adults each rely upon the visual cache and inner scribe mechanisms when performing a spatial-sequential working memory task. As discussed above, one would predict that spatial processing would be employed throughout successful task performance and that a spatial interference task would therefore be disruptive to the span level achieved. However, as current cognitive aging theory predicts less specialized cognitive processing with aging, and more generalized compensatory processing (Reuter-Lorenz and Park, 2014), it is possible that older adults may show a different pattern of interference effects than younger adults. For example, they may show less spatial interference, and/or more interference from the visuo-spatial sketchpad resource which is less specialized for this task (the visual cache). Indeed, Fiore et al. (2012) showed that updating in visuo-spatial working memory is age-sensitive, and suggested that older adults engage in less active rehearsal in spatial working memory than younger adults. This was on the basis of greater age effects at early sequence items, in conjunction with intact recency effects in older adults. On the other hand, Jenkins et al. (2000; see also Jenkins et al., 1999) assessed the effect of a visuo-spatial interference task on performance of a spatial working memory task, in which spatial locations were to be recalled (without sequential order). This research showed that, although capacity was reduced with aging, a visuo-spatial concurrent task (tapping on individual colored locations) was no more disruptive to older than younger adults. However, note that the memory task in these studies was not spatial-sequential, and the interference task was not specific to disrupting either the visual cache or inner scribe components of the visuo-spatial sketchpad. Thus, regarding the functional architecture of the visuo-spatial sketchpad, the hypothesis for the current study was that this may be affected by aging, and that differential visual and/or spatial interference effects may be observed across younger and older adults as a consequence of different processing abilities.

In summary, the first key aim of this research was to establish whether or not the degree of active processing in a spatial-sequential working memory task affects the extent of age-related decline observed. The second aim was to establish whether or not the functional architecture underlying spatial working memory may be subject to age-related change. Specifically, this study investigated whether or not there are age-related differences in the reliance on the visual store and inner scribe mechanisms of working memory.

## Materials and Methods

### Design

This study took the form of a 2 (age group; younger, older) × 2 (recall type; forward, backward – repeated measures) × 3 (interference; control, visual, spatial – between participants) mixed factorial design. Task performance was assessed using a mean span measure of capacity (described below).

### Participants

This study was carried out in accordance with the recom mendations of the Ethics of Research on Human Participants, Glasgow Caledonian University, with written informed consent from all subjects, in accordance with the Declaration of Helsinki. There were 75 younger (18–40 years) and 75 older (64–85 years) participants. The younger group comprised 30 males and 45 females with a mean age of 27.93 (*SD* = 5.98). Their mean number of years of formal education was 17.40 (*SD* = 3.17). The older participants comprised 32 males and 43 females, with a mean age of 73.62 years (*SD* = 6.11) and a mean number of years of education of 11.63 (*SD* = 2.61). The older adults were screened for signs of cognitive impairment, and were required to achieve a score of 25 from the possible 30 in the Mini-Mental State Examination (MMSE; Folstein et al., 1975). The mean MMSE score was 28.15 (*SD* = 1.40). **Table 1** presents the participant demographics by age group and interference condition. None of the participants had carried out the task before, and they each received a small participation fee.

**Table 1:** Means (with standard deviations) for each participant group’s demographic data.

	Younger			Older		
	Control	Visual interference	Spatial interference	Control	Visual interference	Spatial interference
Age	28.16 (±6.26)	28.08 (±5.68)	27.56 (±6.21)	74.44(±6.21)	73.08 (±5.58)	73.33 (±6.67)
Sex (M:F)	10:15	12:13	8:17	9:16	9:16	14:11
Years education	17.36 (±3.11)	17.28 (±3.12)	17.56 (±3.40)	11.84 (±2.58)	11.60 (±2.69)	11.44 (±2.65)
MMSE	-	-	-	28.20 (±1.26)	28.08 (±1.41)	28.16 (±1.57)

### Materials

A version of the Corsi blocks test was used for measuring spatial working memory span. This was a modified version of the Spatial Span subtest of the WMS-III (Wechsler, 1998). The WMS-III Spatial Span board features 10 irregularly spaced blue cubes set upon a white rectangular board, with each cube featuring an identifying number on only the researcher’s side. The board measures approximately 28 cm × 21.5 cm, and the cubes measure 3 cm^3^. The standard task comprises eight sequence levels (ranging from 2 to 9 blocks in length) with two trials at each level. In order to enhance the sensitivity of the task, one additional sequence was created per sequence level, thus allowing three trials at each level. New sequences were generated by adopting a selection without replacement procedure for each of the numbers 1–10. Sequences were then fixed, and each new sequence was administered after the standard two. Consistent with the protocol of the standard Spatial Span test, in the backward recall phase of the task the administration order of the forward phase was reversed within each level, such that the third (new) sequences in each level were administered first. Additionally, so that the sequences were not identical to those administered in the forward recall phase, the order of each sequence was reversed for the backward recall phase, again, as in the standard Spatial Span procedure. Finally, in order to increase working memory demand by requiring the use of rehearsal, the task incorporated a 10 s delay period (retention interval) between presentation and recall.

**FIGURE 1:**
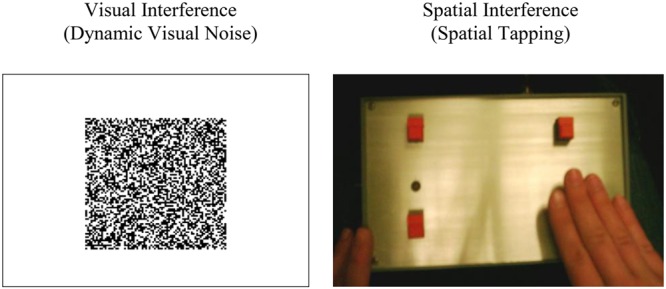
**The visual interference task (dynamic visual noise; **left)** and the spatial interference task (spatial tapping; **right)**.** In the visual task, participants view the dynamically changing array of black and white dots on the computer screen. In the spatial task, participants continually tap around the four locations on the button box in a clockwise direction.

For some participants, either visual or spatial interference took place during the 10 s retention interval of the spatial working memory task. The visual interference took the form of dynamic visual noise (DVN; **Figure 1**), which interferes specifically with the operation of the passive visual store in the visuo-spatial sketchpad (Quinn and McConnell, 1996a,b) and reduces visual imagery and working memory for visual details (e.g., McConnell and Quinn, 2004; Dean et al., 2008; Darling et al., 2009; Borst et al., 2012). The DVN was a computer-generated display of small black and white ‘dots’ which randomly change between black and white in an even and continuous fashion across the array. The array measured 320 pixels × 320 pixels (or approximately 12 cm^2^), and comprised 80 × 80 dots (6400, each 16 pixels in area), which randomly changed between black and white in a continuous, evenly distributed fashion. The rate of change was relatively high, at 30% (1920 dots) per second (McConnell and Quinn, 2000; Dean et al., 2005). The spatial interference was a manual spatial tapping task, with movements to known, predictable locations, which interferes specifically with spatial rehearsal and the inner scribe of working memory, and with minimal central executive involvement (Farmer et al., 1986; Smyth and Pendleton, 1989; Quinn, 1994; Della Sala et al., 1999; Darling et al., 2009). A handheld box was used for this (**Figure 1**), measuring 21.5 cm × 13 cm × 7.5 cm and featuring four buttons (each 1.2 cm × 1.6 cm × 1.1 cm) spaced in a rectangular formation (11 cm × 5.5 cm). An electronic counter was linked to the handheld box for the purpose of calculating the total number of taps per trial.

### Procedure

Participants were randomly allocated to one of the three interference conditions. Each participant sat at a desk opposite the researcher, and the Spatial Span board was positioned between them. From the perspective of the participant, a laptop was positioned to the left of the Spatial Span board. For those in the spatial interference condition, a handheld button box was also placed on the participant’s lap. The first phase of the task measured spatial working memory span with forward recall, and the second phase measured backward recall (as per the standard Spatial Span test; see also Park et al., 2002).

The participant was first instructed to touch the same blocks that the researcher touched, in the same order. Depending on participant choice, one or two practice trials were completed prior to beginning the experimental trials. For a given trial under control conditions, the procedure was as follows: the researcher tapped out the sequence, at a rate of approximately one tap per second, before immediately pressing the button of the mouse, which produced a tone from the laptop; the participant then viewed the blank laptop screen for a period of 10 s, until the word *recall* was presented; the participant then attempted to touch the same blocks in the same order as the researcher. In the visual interference condition, the procedure was the same except that, during the 10 s retention interval, the participant viewed DVN on the screen. In the spatial interference condition, the procedure was the same as in the control condition except that the participant was also required to tap around the buttons on the handheld box in clockwise order with their preferred hand, at their own pace, during the 10 s retention interval. The participant was specifically instructed to view the blank screen and not the handheld box. Some time was provided to allow these participants to familiarize themselves with the spatial tapping task before combining it with the memory task. The electronic counter, which was linked to the box, recorded the number of times the buttons had been tapped within each trial. Regarding memory task performance, the researcher recorded the numbers of the blocks that had been tapped and in which order, and provided performance feedback to the participant. The task continued either until all available trials had been completed, or the participant failed to recall correctly at least one of the three trials from a given level of complexity. Spatial working memory span with forward recall was taken to be the mean size of the last three correctly recalled sequences in this phase.

The second phase of the procedure then measured spatial working memory span with backward recall. The researcher informed participants that the task would be carried out again, except that they were now required to try to reproduce the sequence in the reverse order, beginning with the last cube and working backward. Again, following at least one practice trial, the experimental trials were administered under the same conditions as in forward recall, either until all trials had been administered, or until the participant had failed to recall correctly at least one trial from a given level. The mean size of the last three correctly recalled sequences was calculated^1^.

### Analyses

The *mean span* data were first analyzed using a 2 (age group) × 2 (recall type) × 3 (interference) mixed factorial Analysis of Variance (ANOVA). Follow-up tests were either planned comparisons or Bonferroni-corrected *t*-tests, as appropriate and specified below. Data were then also analyzed using a series of linear regression analyses, in which only the control and spatial interference conditions were included^2^. For the regression analyses, collinearity diagnostics were within acceptable levels [all variance inflation factor (VIF) < 1.47; all tolerance values > 0.68].

## Results

Regarding performance of the spatial interference (tapping) task, during forward recall, the mean number of taps per trial in the younger adults was 15.24 (*SD* = 4.32), and in older adults this was 13.88 (*SD* = 3.15). During backward recall, the mean number of taps in the younger adults was 16.29 (*SD* = 5.08), and in older adults this was 14.60 (*SD* = 3.68). A mixed factorial ANOVA revealed only a significant effect of recall type, *F*(1,48) = 20.37, *MSE* = 0.96, *p* < 0.001, η_p_^2^ = 0.30, in which the number of taps was slightly higher in backward recall (*M* = 15.44, *SD* = 4.47) than in forward recall (*M* = 14.56, *SD* = 3.81). All other effects were not significant (all *p* > 0.19). It is possible that the tapping rate increased slightly with practice. However, it is notable that there were no reliable age effects, and no interaction between the two variables. Particularly as the difference between the two recall types was very small (approximately one tap per trial), tapping rate will therefore not be considered further.

**FIGURE 2:**
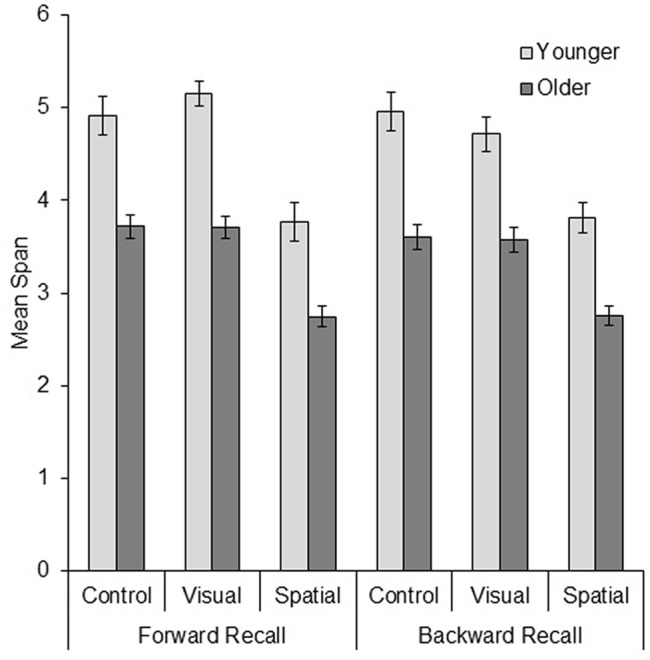
**Mean span data (±SE) from each age group across the three interference conditions, for both forward and backward recall**.

The mean spatial working memory span data are presented in **Figure 2**. The data pattern shows that spatial working memory capacity appears lower for older compared with younger adults, and when the task was carried out alongside the spatial interference condition as compared with both the control and visual interference conditions.

A mixed factorial ANOVA indeed revealed significant effects of age group, *F*(1,144) = 106.12, *MSE* = 1.03, *p* < 0.001, η_p_^2^ = 0.42, with younger adults outperforming older adults, and interference, *F*(2,144) = 33.86, *MSE* = 1.03, *p* < 0.001, η_p_^2^ = 0.32. Follow-up planned comparisons revealed no significant difference between the control and visual interference conditions, *t*(98) = 0.07, *p* = 0.94, but that performance was poorer with spatial than visual interference, *t*(98) = 5.65, *p* < 0.001. There were also non-significant trends for the main effect of recall type, *F*(1,144) = 3.11, *MSE* = 0.228, *p* = 0.080, η_p_^2^ = 0.02, and the interaction between interference and recall type, *F*(2,144) = 2.86, *MSE* = 0.228, *p* = 0.061, η_p_^2^ = 0.04. The interactions between age group and recall type, *F*(1,144) = 0.11, *MSE* = 0.228, *p* = 0.74, η_p_^2^ = 0.001, between age group and interference, *F*(2,144) = 0.50, *MSE* = 1.03, *p* = 0.61, η_p_^2^ = 0.007, and the three-way interaction, *F*(2,144) = 1.49, *MSE* = 0.228, *p* = 0.23, η_p_^2^ = 0.02, were clearly not significant. Bonferroni-corrected paired *t*-tests were used to investigate the trend for the interaction between interference and recall type, analyzing the effect of recall type within each interference condition (with data collapsed across age group). This trend appears to have been driven by an effect of recall type being significant only in the visual interference condition, *t*(49) = 3.23, *p* < 0.01 (all other *p* > 0.72), and may have been influenced by a slightly raised score in the visual interference condition in younger adults’ forward recall.

The ANOVA, then, clearly shows no differential effects of recall type either by interference or age group. To supplement this analysis, however, and to discover the predictors of forward and backward recall performance within each age group, a series of linear regression analyses were then carried out. The data from each age group were therefore analyzed individually, in order to establish which factors were predictive of performance in each age group, using each outcome measure (forward and backward mean span). The relevant correlation matrices are presented in **Tables 2** and **3**. **Table 2** shows that, in younger adults, age is positively related with years of education, as the youngest adults would not yet have completed their education. More interestingly, and in line with the effect of interference described above, the presence of spatial interference was significantly related to lower capacity, in both forward and backward recall. Regarding age, there were non-significant trends for this to be negatively correlated with both forward (*p* = 0.067) and backward (*p* = 0.084) recall. Finally, forward and backward spatial span were positively correlated. **Table 3** highlights that, in older adults, increased age was associated with lower scores on the MMSE. The presence of spatial interference was again significantly related to lower forward and backward span scores in older people, and the two methods of recall were also positively correlated. Backward span was positively related to years of education and MMSE score and, while there was clearly no significant relationship between age and forward span, backward span showed a non-significant trend for a negative association with age (*p* = 0.067).

**Table 2:** Pearson correlation coefficients between each variable included in the younger adult linear regression analyses.

	1	2	3	4	5	6
(1) Age	-	0.19	0.53^∗∗∗^	-0.05	-0.26	-0.25
(2) Sex (0 = M, 1 = F)		-	0.03	0.08	-0.17	-0.13
(3) Years education			-	0.03	-0.25	-0.07
(4) Interference (0 = control, 1 = spatial tapping)				-	-0.49^∗∗∗^	-0.52^∗∗∗^
(5) Mean span – Forward					-	0.76^∗∗∗^
(6) Mean span – Backward						-

*NB: All correlations based upon *N* = 50. ^∗^*p* < 0.05, ^∗∗^*p* < 0.01, ^∗∗∗^*p* < 0.001.*

**Table 3:** Pearson correlation coefficients between each variable included in the older adult linear regression analyses.

	1	2	3	4	5	6	7
(1) Age	-	0.03	-0.05	-0.40^∗∗^	-0.09	-0.05	-0.26
(2) Sex (0 = M, 1 = F)		-	-0.02	-0.08	-0.22	0.11	-0.12
(3) Years education			-	0.16	-0.06	0.16	0.29^∗^
(4) MMSE				-	-0.01	0.25	0.32^∗^
(5) Interference (0 = control, 1 = spatial tapping)					-	-0.64^∗∗∗^	-0.58^∗∗∗^
(6) Mean span – Forward						-	0.68^∗∗∗^
(7) Mean span – Backward							-

*NB: All correlations based upon *N* = 49. ^∗^*p* < 0.05, ^∗∗^*p* < 0.01, ^∗∗∗^*p* < 0.001.*

Linear regression analysis was first carried out on the younger adult data, to establish the predictors of spatial working memory capacity with forward recall. Age, sex, years of education, and interference (control or spatial) were entered into the analysis. The model was significant, *F*(4,45) = 5.80, *p* = 0.001, and predicted 28% of the variance in forward recall (*R* = 0.58, adjusted *R*^2^ = 0.28, *SE* = 1.00). However, as shown in **Table 4**, only interference (β = -0.49, *p* < 0.001) significantly predicted forward recall. For the backward recall data, the model was again significant, *F*(4,45) = 6.34, *p* < 0.001, and predicted 30% of the variance (*R* = 0.60, adjusted *R*^2^ = 0.30, *SE* = 0.93). However, this time, both interference (β = -0.54, *p* < 0.001) as well as age (β = -0.34, *p* = 0.025) served as significant predictors.

**Table 4:** Linear regression models predicting the forward and backward spatial working memory recall of younger and older adults.

		*B*	*SE B*	β	*t(p)*
**Younger**					
Forward	Age	-0.04	0.03	-0.20	-1.36 (0.18)
	Sex	-0.21	0.30	-0.09	-0.71 (0.48)
	Years education	-0.05	0.05	-0.13	-0.90 (0.37)
	Interference	-1.14	0.29	-0.49	-4.01 (<0.001)
Backward	Age	-0.06	0.03	-0.34	-2.32 (0.025)
	Sex	-0.06	0.28	-0.02	-0.20 (0.84)
	Years education	0.04	0.05	0.13	0.89 (0.34)
	Interference	-1.19	0.26	-0.54	-4.50 (<0.001)
**Older**					
Forward	Age	0.00	0.01	-0.004	-0.03 (0.97)
	Sex	-0.03	0.17	-0.02	-0.15 (0.88)
	Years education	0.02	0.03	0.08	0.71 (0.48)
	MMSE	0.12	0.07	0.23	1.88 (0.067)
	Interference	-0.97	0.17	-0.64	-5.65 (<0.001)
Backward	Age	-0.03	0.01	-0.23	-2.07 (0.044)
	Sex	-0.34	0.15	-0.24	-2.32 (0.025)
	Years education	0.06	0.03	0.21	2.09 (0.043)
	MMSE	0.09	0.06	0.17	1.53 (0.134)
	Interference	-0.91	0.15	-0.64	-6.14 (<0.001)

In the older adults, the same variables were entered into a linear regression analysis, along with the additional MMSE variable. Again, the model was significant, *F*(5,43) = 7.99, *p* < 0.001, and predicted 42% of the variance in forward recall (*R* = 0.69, adjusted *R*^2^ = 0.42, *SE* = 0.58). Only interference (β = -0.64, *p* < 0.001) significantly predicted forward recall, although a non-significant trend can be noted in relation to the MMSE scores (β = 0.23, *p* = 0.067). The significant model of the backward recall data, *F*(5,43) = 11.39, *p* < 0.001, predicted 52% of the variance in performance (*R* = 0.76, adjusted *R*^2^ = 0.52, *SE* = 0.50). Both interference (β = -0.64, *p* < 0.001) as well as age (β = -0.23, *p* = 0.044) significantly predicted performance. Thus, in both younger and older adults, age is a significant predictor of, specifically, backward recall. Additionally, however, sex (β = -0.24, *p* = 0.025) and years of education (β = 0.21, *p* = 0.043) also significantly contributed to the model of backward span in older people, suggesting that better performance was associated with males, and a higher number of years of education.

## Discussion

This study investigated spatial-sequential working memory performance in younger and older adults. The working memory task varied according to the more passive (forward) or active (backward) nature of the recall format. Furthermore, interference tasks carried out during the retention interval in the tasks were intended to disrupt temporary visual storage (the operation of the visual cache) or spatial processing (inner scribe functioning). The study was aimed at establishing whether or not aging is associated with a greater decline in active vs. passive spatial working memory, and the extent to which the functional architecture of the visuo-spatial sketchpad is affected by age. Initial analyses showed that recall type did not reliably affect performance either in younger or older adults, and that specialized spatial processing, as indicated by spatial interference effects, appears to be used by both age groups in both passive and active spatial span tasks. However, supplementary linear regression analyses within each age group, which included additional demographic variables, showed that age significantly predicts specifically backward spatial span performance within both younger and older adults, suggesting that the more active recall format is more sensitive to the aging process throughout the adult lifespan.

In terms of the overall effect of aging on spatial working memory capacity, the results clearly showed a reliable age-related deficit, in both forward and backward recall. Robbins et al. (1998) argued that spatial short-term memory performance, as measured by the Corsi with immediate recall, exhibits little decline in older age. In contrast, Moffat et al. (2001; see also Jenkins et al., 2000) concluded that spatial working memory is markedly impaired by the aging process. The present results support the latter suggestion, that spatial working memory capacity is reliably reduced with aging. Note, however, that the task presently employed was designed to place significant demands on spatial working memory, due to the requirement to maintain the sequences over a 10 s delay period before recall. As noted previously, active processing in the form of spatial rehearsal is already necessary for successful performance of a spatial working memory task which features a delay period. However, in the present study, the extent of active processing was directly assessed, in order to compare active rehearsal with the requirement also to manipulate the information prior to recall (Cornoldi and Vecchi, 2003). The latter was expected to draw more heavily upon the resources of the domain-general central executive in the working memory system (Baddeley, 2007; Rudkin et al., 2007; Logie, 2011; Vandierendonck, 2016).

### Passive vs. Active Processing

There has been debate regarding the potential role of relatively active processing in age-related cognitive decline. Previous research has suggested that active tasks, which involve transforming, manipulating, or integrating information (Cornoldi and Vecchi, 2003) are more sensitive to aging (Vecchi and Cornoldi, 1999; Vecchi et al., 2005; Cansino et al., 2013). Indeed, in a meta-analysis, Bopp and Verhaeghen (2005) demonstrated progressively larger age-related deficits depending on the extent of active processing required in a task, from simple (forward) storage span, to backward span, and finally to processing intensive working memory tasks such as sentence span. An interesting finding resulting from the initial ANOVA was that there was no differential effect of aging upon task performance in the forward and backward recall conditions. This supports the results of Hester et al. (2004), who found no interaction between recall type and age in performance of the WMS-III Spatial Span subtest. Additionally, the findings of Vandierendonck et al. (2004) were supported, as they observed no effect of recall type in performance of the Corsi in younger adults. In the present study, the lack of reliable main effect may have been due to the significant maintenance period, which meant that, even in forward recall, the material required active rehearsal to avoid decay prior to the recall stage (Cornoldi and Vecchi, 2003; Baddeley, 2007; Logie, 2011; Vandierendonck, 2016).

However, further linear regression analyses within each age group indicated that the backward recall task may indeed require more active processing than the forward task. Within both age groups, linear regression analyses showed that age significantly predicted backward spatial span, but not forward span. This supports the idea that working memory is vulnerable to decline from early in the adult lifespan, given that age was also predictive of performance within those aged only 18–40 years (Park et al., 2002; Logie and Maylor, 2009; Johnson et al., 2010). Theoretically, the difference between the two tasks was the requirement to draw upon the central executive to re-order the sequences. Thus, specifically central executive functioning may be at least partly responsible for the age-related decline in spatial working memory from early adulthood. Cornoldi and Mammarella (2008) recently argued that forward and backward spatial span differ regarding the underlying resources. They analyzed the effects of recall type across two spatial ability groups, which were categorized as low and high, on the basis of a spatial processing (mental rotation) task. Performance in forward recall was found to be better than in backward recall, but only in the low spatial ability participants, indicating that backward recall does involve additional processing. This additional processing could potentially be more complex spatial processing, given the distinction in these participant groups on the basis of spatial ability. However, this could also be domain-general processing, which their spatial processing task likely also has in common with backward spatial span.

The central executive has been assumed to underlie, at least in part, the processing difference between forward and backward recall. However, particularly as a large amount of variance remained unexplained in the linear regression models, it is important to consider other potential mechanisms underlying the age-related decline in spatial-sequential working memory. Also, Belleville et al. (1998) directly investigated the possibility that central executive manipulation of the contents of working memory may underlie age-related deficits in capacity, and found no evidence for a general central executive deficit in aging. One candidate mechanism is processing speed (Phillips and Hamilton, 2001). Articulatory suppression typically does not affect spatial working memory span (e.g., Vandierendonck et al., 2004), supporting the claim that the task does not rely on verbal working memory. However, Smyth and Scholey (1996) observed that articulation rate reliably predicts spatial working memory span, with or without sequential order, and concluded that the likely source of this shared variance is cognitive processing speed. Certainly, one influential theory of cognitive aging is that processing speed underlies most of the variance in cognitive functioning in older age (Salthouse, 1996), and this has been shown to be important specifically in visuo-spatial cognition in older adults (Brown et al., 2012; Guest et al., 2015). In the context of the present task, processing speed could be crucial to task performance during sequence encoding, manipulation (in the backward recall), rehearsal, and also in the recall stage. Clearly, there are numerous opportunities in the task for slowed processing to affect performance.

In addition to the effect of interference (discussed below), there were two other significant predictors of backward spatial span in older adults; sex and years of education. Although not specifically expected to predict performance in older people, previous research has identified that males are superior to females specifically within an active visuo-spatial working memory task (Vecchi and Girelli, 1998; see also Kaufman, 2007). Cansino et al. (2013) recently used verbal and visuo-spatial *n-back* tasks of different levels of demand (1- or 2-back) to assess the age-related decline in working memory in 1,500 participants across the adult lifespan. Not only did they show that age effects begin as early as in the third decade of life, but the effects begin earliest for more demanding working memory tasks, and the decline begins earlier in women than men for visuo-spatial working memory. Thus, although it is recommended to interpret the present evidence with caution, particularly as the spatial interference condition had slightly more older males than older females, it is interesting that the finding does relate to existing evidence. A further possible source of the age-related decline in spatial-sequential working memory is strategy, when considering the predictive power of years of education in backward recall in older adults (Orsini et al., 1987). This is increasingly being addressed in the cognitive aging literature, in the context of compensation and lifestyle factors that are being taken into account in current influential perspectives (Bailey et al., 2008; Reuter-Lorenz and Park, 2014). As noted earlier, Fiore et al. (2012) suggested that older adults may not use active spatial rehearsal to the same extent as younger adults. Although the present interference effects suggests that, on average, older adults were using active rehearsal, given the overall age-related deficit that was observed, it would be useful to establish whether or not older adults can benefit from strategy training in a spatial-sequential task.

### Functional Architecture of Visuo-Spatial Working Memory

Clear, specific interference effects were observed in both younger and older adults when they were performing the spatial interference task, but not the visual interference task. This provides further evidence that spatial span relies upon spatial processing, and therefore the active spatial rehearsal mechanism of the visuo-spatial sketch pad, but not the visual storage resource within working memory (i.e., visual cache; Logie, 2011; see also Mammarella et al., 2013). Importantly, as both age groups exhibited this effect, the evidence suggests that both age groups typically use the most relevant form of working memory rehearsal when performing the task. This is in line with Jenkins et al. (2000), who administered a visuo-spatial interference task in conjunction with a spatial working memory task and found the same interference effects in both younger and older adults. However, the present results develop upon this previous evidence by having incorporated more specific visual and spatial interference tasks, as opposed to one more general visuo-spatial interference task (see also Jenkins et al., 1999). The evidence also suggests that spatial span does not rely upon the operation of the passive visual store in working memory (Logie, 2011; Mammarella et al., 2013).

In terms of the use of visual and spatial working memory across the two age groups, then, younger and older adults have been shown to use the same spatial strategy which may be assumed to be the most effective one for task completion. Both age groups were shown not to rely upon visual working memory and this is beneficial to overall performance on the task. This is because the retention of a visual image may have allowed for successful recall of the appropriate block locations of a given sequence, but it would not have been conducive to recalling the sequential order. Interestingly, however, there was a potential effect of DVN specifically in the backward recall task, although the interaction between recall type and interference was not reliable. St Clair-Thompson and Allen (2013) investigated the effects of recall type and visual interference (DVN) on digit recall, and found that DVN presented during recall (but not encoding) disrupted specifically backward recall. They argued that visual imagery is a strategy more likely to be used during backward recall. It is therefore possible that the potential effect of DVN on backward recall in the current study is indicative of visual processing being used to some extent to aid with the more challenging backward recall. That is, some participants may have tried to rely upon the visual image of the layout, while manipulating the sequential order, at least in some of the more demanding levels (Vandierendonck et al., 2004). This possibility would be a useful avenue for future research, for understanding the potentially greater involvement of visual store of the visuo-spatial sketchpad in spatial working memory with backward recall, or other more demanding conditions that push working memory beyond capacity limits (Logie, 2011).

In future investigations of the effect of active processing in spatial-sequential working memory, it would be useful to take into account a number of methodological factors that could influence the findings. The present study used the standard spatial span approach by asking participants first to complete the forward task version, followed by the backward recall task (e.g., Park et al., 2002). Rowe et al. (2008) argued that younger adults’ boost in performance under ascending compared with descending sequence presentation formats indicates an important role for practice in spatial span. As younger adults typically begin the task well below their capacity, they gain more practice at the smaller sequence lengths, thus, younger adults’ performance may be overestimated relative to that of older adults, who receive less practice. In the present context, this suggests not only that it would be useful to assess the effect of recall type with a counterbalanced administration procedure, but also that it would be interesting to observe the potential effects of controlling for the extent of task practice. Another issue raised by Wilde and Strauss (2002), is that the standard spatial span task presents the same sequences, but in reverse order, for the backward recall task version. Although rather unlikely, it is possible that participants may store memory traces of the stimuli, particularly if the first task version does not progress very far, which can often be the case in older adults. Thus, administering entirely new sequences in the backward span task would be useful in order to control for this potential issue.

## Conclusion

This research has shown that spatial-sequential working memory is subject to age-related decline. Although a subject of debate, backward recall, which is assumed to require more active, central executive processing, does appear to be more sensitive to aging in spatial working memory, in the context that age significantly predicted backward recall performance within both the younger and older adult groups. Additionally, the functional architecture of the visuo-spatial sketchpad was shown not to be affected by age when performing forward and backward spatial-sequential working memory tasks, at least in the present conditions. Both age groups were shown to rely upon the most appropriate, specialized processing for task completion (active spatial rehearsal), as a spatial interference task exhibited specific interference effects in both age groups, across both the passive and active recall task versions. While younger and older adults appear to engage in active spatial rehearsal during a spatial-sequential span task, backward spatial span may indeed offer a more sensitive measure of spatial working memory performance across the adult lifespan.

## Author Contributions

LB is responsible for designing this research, acquiring, analyzing, and interpreting the data, drafting the manuscript, and is accountable for all aspects of the work.

## Conflict of Interest Statement

The author declares that the research was conducted in the absence of any commercial or financial relationships that could be construed as a potential conflict of interest.

## Footnotes

1 For 4 of the 25 older participants in the spatial interference condition in forward recall, and 6 of the 25 older participants in the spatial interference condition in backward recall, only one or two successful trials had taken place. In these cases, the mean was taken of the available successful trials.

2 However, the same pattern of findings is observed when including the visual interference condition data (collapsed with the control condition). Note also that, for the linear regression analyses, the older adult group was reduced from 50 to 49 participants, due to one missing datapoint.
